# Glial D-serine modulates oligodendrocyte lineage progression under inflammatory conditions

**DOI:** 10.3389/fncel.2026.1784678

**Published:** 2026-03-16

**Authors:** Juan Pablo Espinoza, Ignacio Cisterna, Juan Jose Triviño, Verónica Arancibia, Sebastián Beltrán-Castillo

**Affiliations:** 1 Centro Integrativo de Biología y Química Aplicada (CIBQA), Universidad Bernardo O’Higgins, Santiago, Chile; 2 Doctorado en Ciencias Mención Materiales Funcionales, Facultad de Ciencias de la Salud, Universidad Bernardo O’Higgins, Santiago, Chile; 3 Facultad de Ciencias Químicas y Farmacéuticas, Universidad de Chile, Santiago, Chile

**Keywords:** astrocytes and microglia, d-serine, glial signaling, neuroinflammation, NMDA receptors, oligodendrocyte differentiation, oligodendrocyte precursor cells, remyelination

## Abstract

Inflammatory environments may shape oligodendrocyte lineage dynamics beyond classical cytokine signaling, in part through the release of glial neuromodulators. Here, we investigated whether inflammation-associated D-serine signaling modulates oligodendrocyte lineage progression. Using highly purified primary oligodendrocyte precursor cell (OPC) cultures, we show that D-serine exposure during late differentiation reduces the proportion of OLIG2^+^ and myelin basic protein–positive (MBP^+^) cells without altering net cell number, while selectively decreasing apoptosis within the mature MBP^+^ population. These findings indicate that D-serine attenuates late-stage lineage progression while preserving oligodendrocyte survival *in vitro*. In parallel, inflammatory activation of mixed glial cultures with lipopolysaccharide (LPS) increased tumor necrosis factor-*α* release, upregulated serine racemase expression, and elevated extracellular D-serine levels. Conditioned media from reactive glial cultures recapitulated the effects of D-serine on OPC maturation, which were prevented by enzymatic degradation of D-serine or pharmacological blockade of N-methyl-D-aspartate receptors (NMDARs). Together, these findings support the involvement of glia-derived D-serine as a modulatory signal influencing oligodendrocyte lineage progression consistent with NMDAR-dependent mechanisms under inflammatory conditions that may contribute to impaired remyelination.

## 1 Introduction

Oligodendrocytes (OLs) are the cells responsible for ensheathing neuronal axons in the central nervous system (CNS) with myelin, thereby enabling fast saltatory conduction (Fünfschilling et al., 2012; Simons and Nave, 2015; Duncan et al., 2021). The loss or dysfunction of OLs compromises axonal integrity and neural communication, a hallmark of demyelinating disorders such as multiple sclerosis (MS). The most common clinical form, relapsing–remitting MS (RRMS), affects approximately 80%–85% of patients and is characterized by inflammatory relapses that promote OL damage and axonal degeneration, followed by remission phases during which partial functional recovery occurs, through plasticity and limited remyelination, within a residual inflammatory environment. Over time, incomplete repair leads to cumulative pathology and persistent neurological deficits, including motor weakness and sensory disturbances.

Current MS therapies primarily target immune activation to reduce relapse frequency and delay the progression of irreversible neurological disability (Rommer et al., 2019). However, enhancing remyelination during remission represents an additional therapeutic opportunity to promote functional recovery. Remyelination relies in part on newly generated OLs derived from oligodendrocyte precursor cells (OPCs), a widespread and dynamic population that persists throughout adulthood and serves as a reservoir for oligodendrocyte replacement (Dimou et al., 2008; Rivers et al., 2008; Young et al., 2013). Thus, understanding the signals that regulate OPC maintenance and their progression toward mature, myelin-forming OLs is critical for improving remyelination efficiency, particularly in the context of demyelinating lesions.

The ability of OPCs to respond to demyelinating injury is strongly shaped by the inflammatory milieu (Zveik et al., 2024), which is orchestrated by resident glial cells, particularly microglia and astrocytes. Upon activation, these cells release a broad spectrum of cytokines, reactive oxygen species, and other signaling molecules that remodel the local microenvironment and influence OPC survival and differentiation (McTigue and Tripathi, 2008; French et al., 2009; Miron et al., 2013; Hammond et al., 2014; Franklin and Ffrench-Constant, 2017; Miron, 2017). Beyond classical inflammatory mediators, accumulating evidence indicates that inflammatory activation enhances the release of D-serine from both microglia and astrocytes (Wu et al., 2004, 2007; Perez et al., 2017; Beltrán-Castillo et al., 2020; Tapanes et al., 2022).

D-serine is a D-amino acid synthesized in the CNS through the racemization of L-serine by serine racemase (SR) (Wolosker et al., 1999a, 1999b) and functions as a co-agonist at the glycine-binding site of N-methyl-D-aspartate receptors (NMDARs). Multiple NMDAR subunits, including GluN1, GluN2A–D, and GluN3A, are expressed across the oligodendrocyte lineage, and functional NMDAR-mediated currents have been recorded in both OPCs and differentiated OLs (Káradóttir et al., 2005; Salter and Fern, 2005; Micu et al., 2006; Li et al., 2013). These observations suggest that oligodendroglial cell functions are sensitive to D-serine levels, likely through NMDAR-dependent mechanisms. However, while emerging evidence supports roles for oligodendroglial NMDARs in axonal metabolic support (Krasnow and Attwell, 2016; Saab et al., 2016) and in pathways associated with OPC differentiation, their functional significance remains incompletely understood, particularly under inflammatory conditions. Whether glia-derived D-serine acts on oligodendroglial NMDARs to modulate lineage progression during inflammation therefore remains unresolved.

In this study, we examined whether D-serine released from activated glial cells modulates OPC behavior under inflammatory conditions. Using primary OPC cultures, we assessed the effects of D-serine on cell density, survival, and differentiation, and evaluated whether conditioned media from inflammatory glial cultures could recapitulate these effects in a manner sensitive to pharmacological NMDAR blockade. Our findings support the involvement of inflammation-associated D-serine signaling as a modulatory pathway shaping oligodendroglial lineage progression, with potential implications for remyelination efficiency in inflammatory demyelinating disorders.

## 2 Materials and methods

### 2.1 Animals

Postnatal day 0–3 (P0–P3) C57BL/6J wild-type mice were obtained from the animal facility of Universidad Bernardo O’Higgins (UBO). All animal procedures were conducted in accordance with the National Institutes of Health Guide for the Care and Use of Laboratory Animals (NIH Publication No. 8023, revised 1978) and the Bioethical Guidelines for Research Involving Animals of the Chilean National Agency for Research and Development (ANID). All experimental protocols were approved by the Institutional Ethics Committee of Universidad Bernardo O’Higgins.

### 2.2 Cell suspension

Mouse pups (P0–P3) were euthanized by rapid decapitation, and the cerebral cortices were rapidly dissected and transferred to ice-cold calcium-, magnesium-, and phenol red–free Hanks’ Balanced Salt Solution (HBSS; Gibco, Cat. No. 14175-095). After removal of the meninges, cortices were rinsed three times in HBSS and minced into small pieces. Tissue fragments were incubated with 0.05% trypsin–EDTA (1X; Gibco, Cat. No. 25300-054) at 37 °C for 30 min. Following enzymatic digestion, cells were mechanically dissociated by gentle trituration (20 passes) using a fire-polished glass Pasteur pipette in Basal Medium Eagle (BME; Gibco, Cat. No. 21010-046) supplemented with 10% horse serum (HyClone™, Cat. No. SH30074-03), GlutaMAX™ (100X; Gibco, Cat. No. 35050-061), and penicillin–streptomycin (Gibco, Cat. No. 15070-063). The resulting cell suspension was filtered through a 70 μm cell strainer, washed with HBSS, and centrifuged at 300 × *g* for 10 min at 4 °C.

### 2.3 OPC isolation by magnetic-activated cell sorting (MACS)

Oligodendrocyte precursor cells (OPCs) were isolated by positive magnetic-activated cell sorting using CD140a (PDGFRα) MicroBeads (Miltenyi Biotec, Cat. No. 130-101-502), according to the manufacturer’s instructions. Briefly, the cell pellet was resuspended in PB buffer (PBS, pH 7.2, supplemented with 0.5% bovine serum albumin) and incubated with Fc receptor (FcR) blocking reagent for 10 min at 4 °C. Cells were then incubated with CD140a (PDGFRα) MicroBeads for 15 min at 4 °C. After washing with PB buffer, cells were centrifuged at 300 × *g* for 10 min at 4 °C and resuspended in fresh PB buffer. The cell suspension was applied to a MACS® separation column placed in a magnetic field (Miltenyi Biotec), allowing the isolation of the magnetically labeled CD140a^+^ OPC fraction and the unlabeled fraction for subsequent culture.

### 2.4 Cell culture conditions

CD140a^+^ cells (OPCs) retained during positive magnetic sorting were seeded at a density of 20,000 cells per well in 24-well plates onto sterile glass coverslips previously coated overnight with 0.01% poly-L-lysine (Sigma-Aldrich) at 37 °C. OPCs were cultured in MACS Neuro Medium (Miltenyi Biotec) supplemented with 2% MACS NeuroBrew®-21 (Miltenyi Biotec), 1% penicillin–streptomycin (Gibco, Cat. No. 15070-063), 0.5 mM GlutaMAX™ (Gibco, Cat. No. 35050-061), 10 ng/mL human platelet-derived growth factor AA (PDGF-AA; Miltenyi Biotec, Cat. No. 130-093-977), and 10 ng/mL human fibroblast growth factor-2 (FGF-2; Miltenyi Biotec, Cat. No. 130-093-837). Each well of the 24-well plate contained 500 μL of total culture medium.

Non-retained cells were seeded into standard 6-well plates and cultured in DMEM/F12 medium (PAN-Biotech) supplemented with 10% fetal bovine serum (FBS; Cytiva) and 1% penicillin–streptomycin. Under these conditions, non-retained cells expanded as mixed glial cultures, a system that predominantly supports astrocyte proliferation while maintaining a smaller microglial population. Mixed glial cultures were prepared from neonatal cortical tissue following careful removal of meninges, minimizing fibroblast contamination, and were expanded under serum-containing conditions. All cultures were maintained at 37 °C in a humidified incubator with 5% CO₂, and the culture medium was replaced every 48 h.

### 2.5 Glial-conditioned medium (CM) generation

Mixed glial cultures were used as a source of glial-conditioned media to investigate the effects of inflammation-induced D-serine release on OPC behavior. Glial identity was confirmed by immunocytochemistry, showing abundant GFAP^+^ astrocytes and IBA1^+^ microglia. When cultures reached approximately 70% confluency, cells were washed with PBS and incubated in serum-free DMEM/F12 medium at 37 °C for 30 min to remove residual serum. Lipopolysaccharide (LPS; 1 μg/mL; Sigma-Aldrich, Cat. No. L3129) was then added for 1 h to induce inflammatory activation.

In selected experiments, extracellular D-serine levels were modulated by pre-treating cultures for 1 h with D-amino acid oxidase (DAAO; 0.1 U/mL; Sigma-Aldrich, Cat. No. A5222-100 U) and catalase (260 U/mL; Sigma-Aldrich, Cat. No. C40-100MG), followed by co-application during LPS exposure. After treatments, the medium was replaced with OPC differentiation medium lacking mitogenic factors (PDGF-AA and FGF-2), and DAAO plus catalase were re-applied where indicated. Cultures were maintained for an additional 16 h under standard conditions (37 °C, 5% CO₂), after which the conditioned medium (CM) was collected, centrifuged to remove cellular debris (300 × *g* for 10 min at 4 °C), and stored at −80 °C until further use.

### 2.6 OPC treatments

On day *in vitro* 6 (DIV6), OPC cultures were washed with PBS and exposed to the assigned experimental treatments. For each treatment condition, the existing medium was completely replaced with 500 μL of the corresponding treatment solution. Cells were treated with conditioned medium (CM) obtained from mixed glial cultures, either unstimulated or stimulated with lipopolysaccharide (LPS), and supplemented, where indicated, with D-amino acid oxidase (DAAO) and catalase. In parallel experiments, OPCs were treated directly with D-serine (1–10 μM, as indicated) or with the non-competitive N-methyl-D-aspartate receptor (NMDAR) antagonist (+)-MK-801 maleate (10 μM; Tocris Bioscience, Bio-Techne, Cat. No. 0924). For all CM-based treatments, platelet-derived growth factor AA (PDGF-AA; 10 ng/mL) and fibroblast growth factor-2 (FGF-2; 10 ng/mL) were included to maintain OPC viability. After 16 h under standard culture conditions (37 °C, 5% CO₂), cells were washed with PBS and fixed for subsequent analyses.

### 2.7 Immunocytochemistry, TUNEL assay, and cell quantification

To assess cell death in specific oligodendrocyte populations, terminal deoxynucleotidyl transferase dUTP nick-end labeling (TUNEL) staining was performed in combination with immunocytochemistry for lineage-specific markers. The following primary antibodies were used to identify distinct stages of the oligodendrocyte lineage: platelet-derived growth factor receptor alpha (PDGFRα; rabbit monoclonal, clone D13C6 XP®, Alexa Fluor® 488–conjugated, Cell Signaling Technology, Cat. No. 8871, 1:200) for OPCs; Olig2 (goat polyclonal, R&D Systems, Cat. No. AF2418, 5–15 μg/mL) for oligodendroglial lineage cells; and myelin basic protein (MBP; rabbit monoclonal, clone D8X4Q XP®, Cell Signaling Technology, Cat. No. 78896, 1:50) for mature oligodendrocytes.

Cells were fixed with freshly prepared 4% paraformaldehyde (PFA) in PBS for 30 min at room temperature, washed three times with PBS, and permeabilized with 0.1% Triton X-100 on ice for 2 min. After three additional PBS washes, TUNEL labeling was performed using the *In Situ* Cell Death Detection Kit, TMR Red (Roche), with coverslips incubated in the reaction mixture for 1 h at 37 °C in the dark.

Following TUNEL staining, cells were blocked for 1 h at room temperature in a solution containing 5% donkey serum and 0.1% Triton X-100 in PBS. Primary antibodies or directly fluorophore-conjugated antibodies were applied overnight at 4 °C in a humidified chamber. After three PBS washes, appropriate fluorophore-conjugated secondary antibodies were incubated for 1 h at room temperature where required. Coverslips were then washed and mounted using Fluoromount-G (SouthernBiotech) for imaging.

Fluorescence images were acquired using a Zeiss AxioScope 7 epifluorescence microscope equipped with an Axiocam 202 mono camera and controlled using ZEN 3.2 (blue edition) software (Zeiss). For each experimental condition, 6–8 randomly selected fields (~0.41 mm^2^) were imaged per coverslip under identical acquisition settings. Image acquisition and quantification were performed by an investigator blinded to the treatment conditions. Total DAPI^+^ nuclei per field were used as an estimate of net cell number. Data were expressed as the percentage of marker-positive cells relative to the total number of DAPI-stained nuclei or normalized to control conditions, as indicated.

### 2.8 Western blot

Proteins were extracted using radioimmunoprecipitation assay (RIPA) buffer (Pierce™, Thermo Scientific™) supplemented with a protease inhibitor cocktail (Thermo Scientific™). Cells were lysed by mechanical scraping followed by sonication, and protein concentration was determined using the Micro BCA Protein Assay Kit (Thermo Scientific™). Equal amounts of protein were separated by 10% SDS–polyacrylamide gel electrophoresis (SDS–PAGE) and transferred onto polyvinylidene difluoride (PVDF) membranes (Thermo Scientific™) using a wet transfer system. Membranes were blocked with 3% bovine serum albumin (BSA) in Tris-buffered saline (TBS) for 1 h at room temperature and incubated overnight at 4 °C with primary antibodies (anti–serine racemase, D5V9Z, Cell Signaling Technology, #13285; anti-*β*-Tubulin I/II, JDR.3B8, Sigma-Aldrich, T8535) diluted in 3% BSA in TBS. After three washes with TBS containing 0.1% Tween-20 (TBS-T), membranes were incubated with horseradish peroxidase (HRP)-conjugated secondary antibodies for 1 h at room temperature. Signal detection was performed using an enhanced chemiluminescence (ECL) substrate (Cyanagen), and images were acquired using a UVITEC imaging system (Cambridge, UK). Densitometric analyses were performed using ImageJ software, and protein levels were normalized to β-tubulin.

### 2.9 TNF-α quantification

Tumor necrosis factor-alpha (TNF-α) levels in conditioned media were quantified using the Mouse TNF-α Uncoated ELISA Kit (Thermo Fisher Scientific, Cat. No. 88-7324), according to the manufacturer’s instructions. Briefly, high-binding 96-well plates were coated overnight at 4 °C with the capture antibody diluted in coating buffer, washed, and blocked for 1 h at room temperature. Samples and standards were loaded in duplicate and incubated for 2 h at room temperature with gentle shaking. After washing, the biotin-conjugated detection antibody was applied for 1 h, followed by incubation with streptavidin–horseradish peroxidase (HRP) for 30 min. Colorimetric detection was performed using tetramethylbenzidine (TMB) substrate, and the reaction was stopped with 1 M phosphoric acid. Optical density was measured at 450 nm with wavelength correction at 570 nm using an Infinite® M200 PRO microplate reader (Tecan). TNF-α concentrations were calculated from a standard curve generated with recombinant mouse TNF-α provided with the kit.

### 2.10 D-serine quantification by HPLC-FLD

D-serine levels were quantified by high-performance liquid chromatography with fluorescence detection (HPLC-FLD), as previously described by Hashimoto et al. (1992), with minor modifications. Analyses were performed using an HPLC system equipped with a VWR Hitachi Elite LaChrom 2130 series pump, a manual injection valve fitted with a 20 μL loop, and a 2,455 series fluorescence detector.

Free amino acids were derivatized prior to injection using o-phthalaldehyde (OPA) and N-tert-butyloxycarbonyl-L-cysteine (Boc-L-Cys), generating diastereomeric derivatives that allowed chromatographic separation of D-serine and L-serine. Samples were separated on a reverse-phase ZORBAX Eclipse Plus C18 column (4.6 mm × 100 mm, 3.5 μm) using a mobile phase composed of acetate buffer, acetonitrile, and tetrahydrofuran. The fluorescence detector was set to an excitation wavelength of 344 nm and an emission wavelength of 443 nm.

Calibration curves were prepared in culture medium matrix (DMEM/F12) using D-serine standards (Sigma-Aldrich), yielding a linear range between 0.26 and 5.26 μmol/L. Under these conditions, retention times were approximately 13.0 min for D-serine and 11.3 min for L-serine. Quantification of D-serine in experimental samples was performed using the standard addition method. Final concentrations were normalized to the total protein content of each sample.

### 2.11 Statistical analysis

All data are presented as mean ± SEM. For all experiments, *n* refers to independent biological replicates, each obtained from a distinct primary culture generated from a different pool of neonatal cortices. Thus, all statistical analyses were performed on biologically independent samples. Statistical analyses were conducted using GraphPad Prism 10 (GraphPad Software). Because datasets did not meet assumptions required for parametric testing, non-parametric analyses were used throughout. Multiple-group comparisons were performed using the Kruskal–Wallis test followed by Dunn’s *post hoc* test. Paired comparisons were analyzed using the Wilcoxon matched-pairs signed-rank test. Statistical significance was defined as *p* < 0.05.

## 3 Results

### 3.1 Primary OPC cultures exhibit a temporal progression toward oligodendrocyte maturation

**Figure 1:**
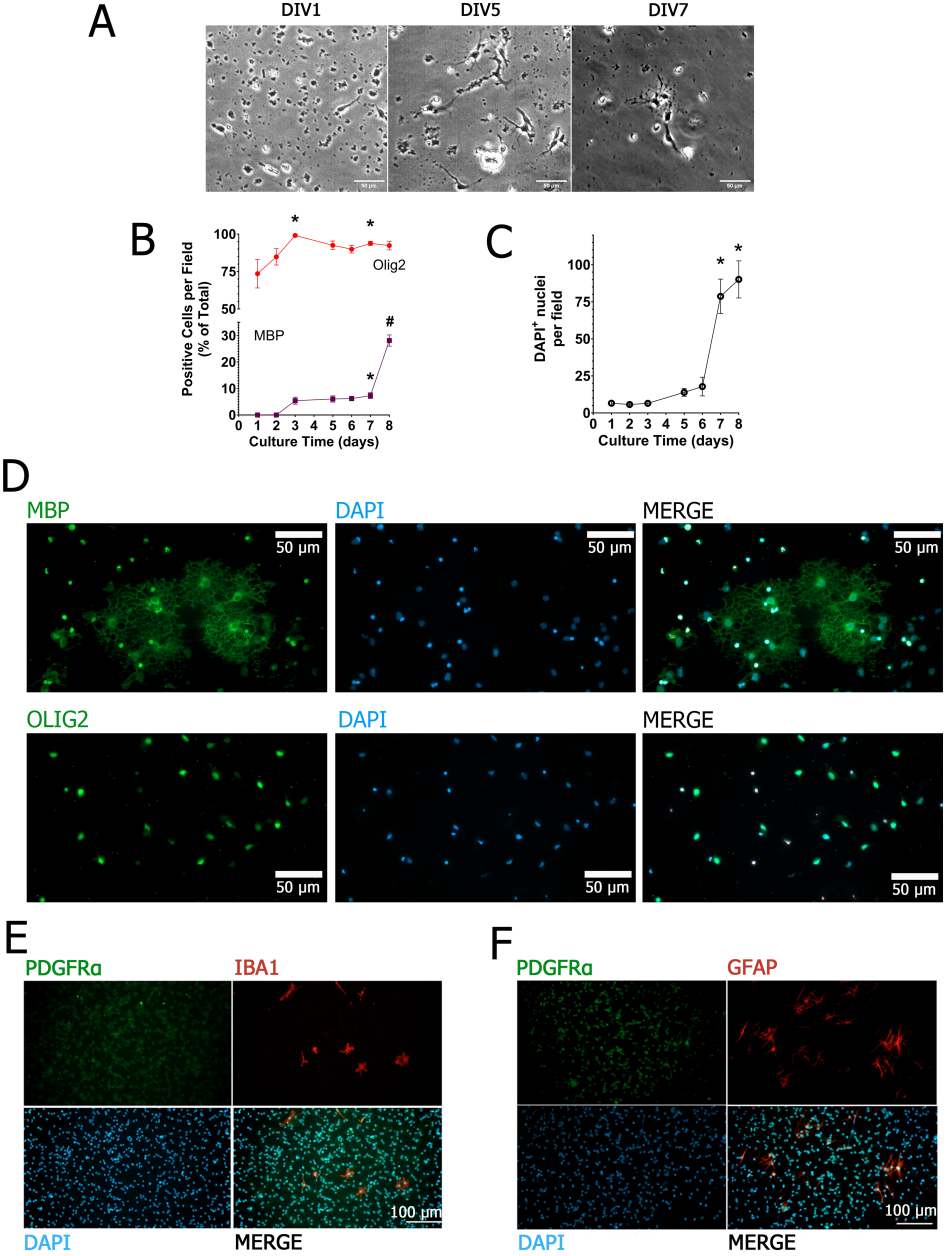
Temporal progression and cellular composition of primary OPC cultures. Phase-contrast images illustrate OPC morphology at DIV1, DIV5, and DIV7 **(A)**. Quantification of OLIG2^+^ and MBP^+^ cells across days *in vitro* (DIV1–DIV7), expressed as percentage of total DAPI^+^ nuclei per field, is shown in **(B)**, while total DAPI^+^ nuclei per field were quantified over time as an estimate of net cell number **(C)**. Representative immunofluorescence images at DIV7 display OLIG2^+^ or MBP^+^ cells (green) with DAPI counterstaining (blue) **(D)**. Culture purity was evaluated at DIV7 by immunostaining for PDGFRα (green) together with microglial (IBA1; panel **E**, red) or astrocytic markers (GFAP; panel **F**, red). Representative fields are shown to illustrate residual non-oligodendroglial cells; quantitative analysis across multiple fields confirmed minimal microglial and astrocytic contamination (<1% of total DAPI^+^ nuclei). Data represent mean ± SEM from seven independent cultures derived from pooled neonatal cortices. Scale bars: 50 μm **(A,D)**; 100 μm **(E,F)**.

To validate the identity and differentiation potential of our primary OPC cultures, we performed a combination of morphological assessments, immunocytochemical analyses, and lineage-marker quantification across multiple time points. Under our culture conditions, OPCs maintained a high net cell number while progressively advancing along the oligodendroglial lineage toward more mature phenotypes. Phase-contrast microscopy (Figure 1A) revealed early bipolar morphology with limited process extension at day 1 *in vitro* (DIV1). Over the following days, cells began to exhibit branched processes indicative of early differentiation (DIV5), and by DIV7 cells displayed increasingly complex, ramified morphologies consistent with progressive maturation.

To further characterize this maturation progression, we analyzed the expression of oligodendrocyte transcription factor 2 (OLIG2) and myelin basic protein (MBP), two well-established oligodendrocyte lineage markers, across multiple DIVs (Figure 1B). OLIG2 is a basic helix–loop–helix (bHLH) transcription factor required for OPC formation and differentiation into OLs (Zhou et al., 2000), whereas MBP is a marker of mature, myelinating OLs (Boggs, 2006). At DIV1, 73.6% ± 9.5% of cells were OLIG2^+^, with no detectable MBP^+^ cells. By DIV3, MBP expression appeared in a small proportion of cells (5.4% ± 1.3%), while OLIG2 labeling remained high (>90%). This trend continued over time, with MBP^+^ cells increasing to 6.1% ± 1.1% at DIV5, 6.2% ± 0.9% at DIV6, 7.3% ± 1.1% at DIV7, and 28.1% ± 2.1% by DIV8. Throughout this period, OLIG2^+^ cells remained the predominant population, indicating preservation of lineage identity during differentiation. In parallel, we quantified DAPI-stained nuclei per field across DIV1–DIV8 as an estimate of net cell number. Cell density increased progressively (Figure 1C), consistent with a progressive increase in net cell number under our culture conditions. Together, these observations indicate that OPCs progressively increase in net cell number and mature over time. Representative immunofluorescence images further support these quantitative findings. At DIV7 (Figure 1D), MBP^+^ cells displayed elongated, branched processes consistent with a differentiated oligodendrocyte morphology, while OLIG2^+^ cells remained abundant within the same cultures, indicating preservation of an oligodendroglial population. At DIV7, immunostaining for IBA1 and GFAP revealed only occasional microglia or astrocytes, which together accounted for <1% of total cells when quantified across multiple fields, although isolated fields containing these cells were occasionally observed (Figures 1E,F). This indicates that our preparations were highly enriched for oligodendroglial lineage cells with minimal interference from other glial types. Taken together, these results demonstrate that our culture system yields highly pure, proliferative OPCs capable of undergoing progressive and temporally regulated differentiation toward mature OLs. This model provides a robust platform to evaluate the influence of inflammatory stimuli and neuromodulators on oligodendroglial lineage dynamics.

### 3.2 D-serine attenuates OPC maturation

**Figure 2:**
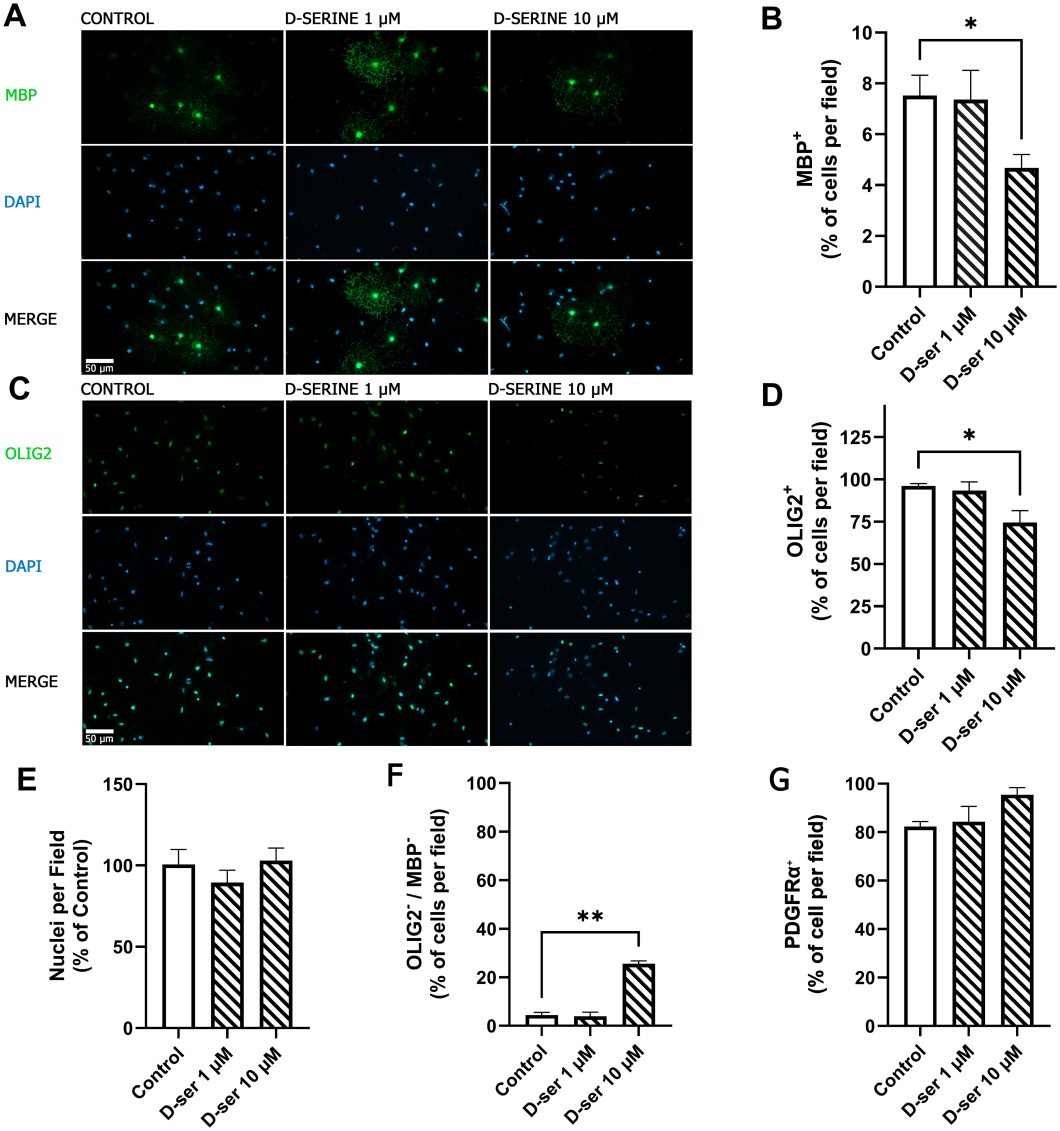
D-serine attenuates late-stage oligodendrocyte lineage progression without altering net cell number. Representative immunofluorescence images of OPC cultures treated with D-serine (1 or 10 μM) at DIV6 and analyzed at DIV7 are shown for MBP **(A)** and OLIG2 **(C)**, with DAPI used to label nuclei. Merged images illustrate lineage marker distribution across conditions. The proportions of MBP^+^ **(B)** and OLIG2^+^ cells **(D)** are expressed as a percentage of total DAPI^+^ nuclei per field. **(E)** Total DAPI^+^ nuclei per field are shown normalized to control values and used as an estimate of net cell number. **(F)** The proportion of OLIG2^−^/MBP^−^ cells and **(G)** the proportion of PDGFRα^+^ cells are expressed as a percentage of total DAPI^+^ nuclei per field. Bars represent mean ± SEM from 6 to 10 independent cultures derived from pooled neonatal cortices. Statistical comparisons were performed using the Kruskal–Wallis test followed by Dunn’s *post hoc* test. *p < 0.05; **p < 0.01.

Given the temporal progression described above, we next examined whether acute D-serine exposure could modulate late-stage OPC progression in our cultures. Based on the maturation dynamics, we selected DIV6 as the intervention point and DIV7 as the evaluation day. Although the most pronounced increase in MBP^+^ cells occurred between DIV7 and DIV8, the first significant increase was detected between DIV6 and DIV7. Thus, DIV6 represents a transitional stage during which lineage progression remains plastic, allowing us to assess whether D-serine modulates maturation prior to the marked expansion of terminally differentiated MBP^+^ cells observed at DIV8. Exposure to D-serine (10 μM) significantly reduced the proportion of MBP^+^ cells compared to control conditions (7.5% ± 0.8% vs. 4.7% ± 0.5%; Figures 2A,B) and was accompanied by a decrease in the proportion of OLIG2^+^ cells (96.2% ± 1.3% vs. 74.6% ± 6.9%; Figures 2 C,D). In contrast, the total number of DAPI^+^ nuclei per field remained unchanged across conditions (Figure 2E), indicating no detectable effect on net cell number. Consistent with the reduction in OLIG2^+^ and MBP^+^ fractions, D-serine treatment resulted in a significant increase in the OLIG2^−^/MBP^−^ fraction (4.4% ± 1.1% vs. 25.6% ± 1.2%; Figure 2F). Because this category was defined by the absence of OLIG2 and MBP staining, it does not by itself establish a specific differentiation identity but rather reflects a shift in lineage-marker distribution within the culture. Notably, the proportion of PDGFRα^+^ cells remained high under all conditions (82.3% ± 2.0% in control vs. 95.4% ± 2.8% at 10 μM D-serine; Figure 2G), indicating preservation of a substantial OPC population across treatments and suggesting that the observed changes primarily reflect shifts in differentiation state rather than cell loss. Collectively, these findings indicate that D-serine attenuates oligodendrocyte lineage progression at late stages of differentiation without altering overall cell density.

### 3.3 D-serine reduces apoptosis of mature MBP^+^ cells

**Figure 3:**
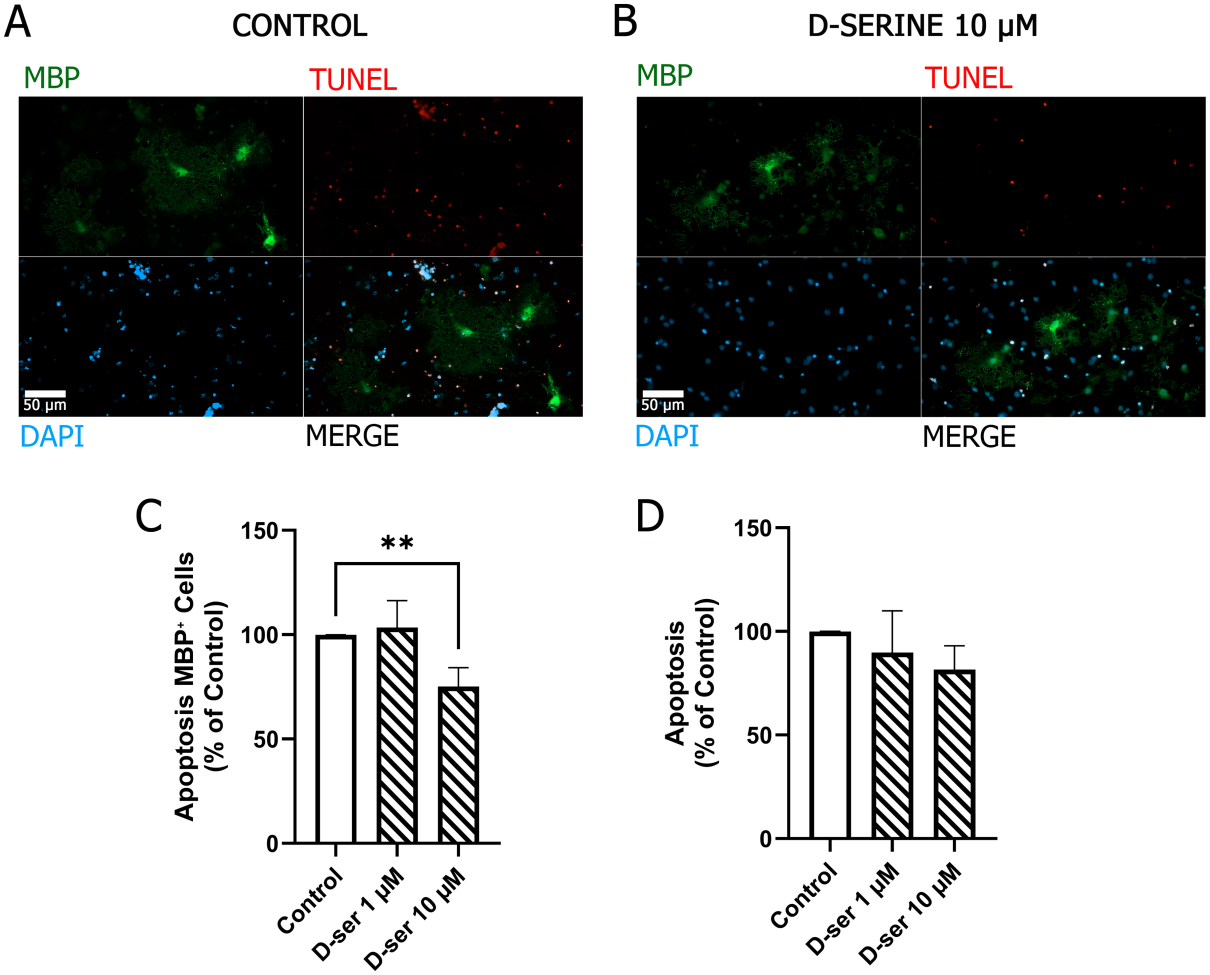
D-serine selectively reduces apoptosis within MBP^+^ cells without altering total apoptosis. OPC cultures were treated with D-serine (1 or 10 μM) at DIV6 and analyzed 24 h later (DIV7). Apoptotic cells were identified by TUNEL staining. **(A,B)** Representative immunofluorescence images of MBP (green), TUNEL (red), and DAPI (blue) in control cultures and cultures treated with 10 μM D-serine. **(C)** Quantification of apoptosis within the MBP^+^ cell population, expressed relative to control values. **(D)** Total apoptosis across all cells, expressed relative to control. In control cultures, apoptotic DAPI^+^ nuclei represented 14.9% ± 1.6% of total DAPI^+^ nuclei; this baseline value was set to 100% for graphical representation. Bars represent mean ± SEM from 6 to 10 independent cultures derived from pooled neonatal cortices. Statistical comparisons were performed using the Kruskal–Wallis test followed by Dunn’s *post hoc* test. *p < 0.05.

To further characterize the effects of D-serine on oligodendrocyte lineage dynamics, we evaluated apoptosis at DIV7 following treatment with 1 or 10 μM D-serine at DIV6. Apoptotic cells were identified using TUNEL staining. Under control conditions, the overall proportion of apoptotic cells in the cultures was 14.9% ± 1.6%, and D-serine treatment did not significantly alter this global apoptosis rate (Figure 3D). In contrast, lineage-specific analysis focusing on the MBP^+^ population revealed a significant reduction in apoptosis following exposure to 10 μM D-serine (Figures 3A,B,C), corresponding to 75.2% ± 9.0% of control levels. In absolute terms, 11.3% ± 3.7% of MBP^+^ cells were apoptotic under control conditions, whereas D-serine treatment reduced this proportion to 7.7% ± 2.7%. No significant changes were detected at the lower concentration. Together, these results suggest that D-serine reduces apoptosis within the mature oligodendrocyte population without altering overall cell death rates in the culture.

### 3.4 LPS-induced reactive glial state is associated with increased serine racemase abundance and elevated D-serine levels

**Figure 4:**
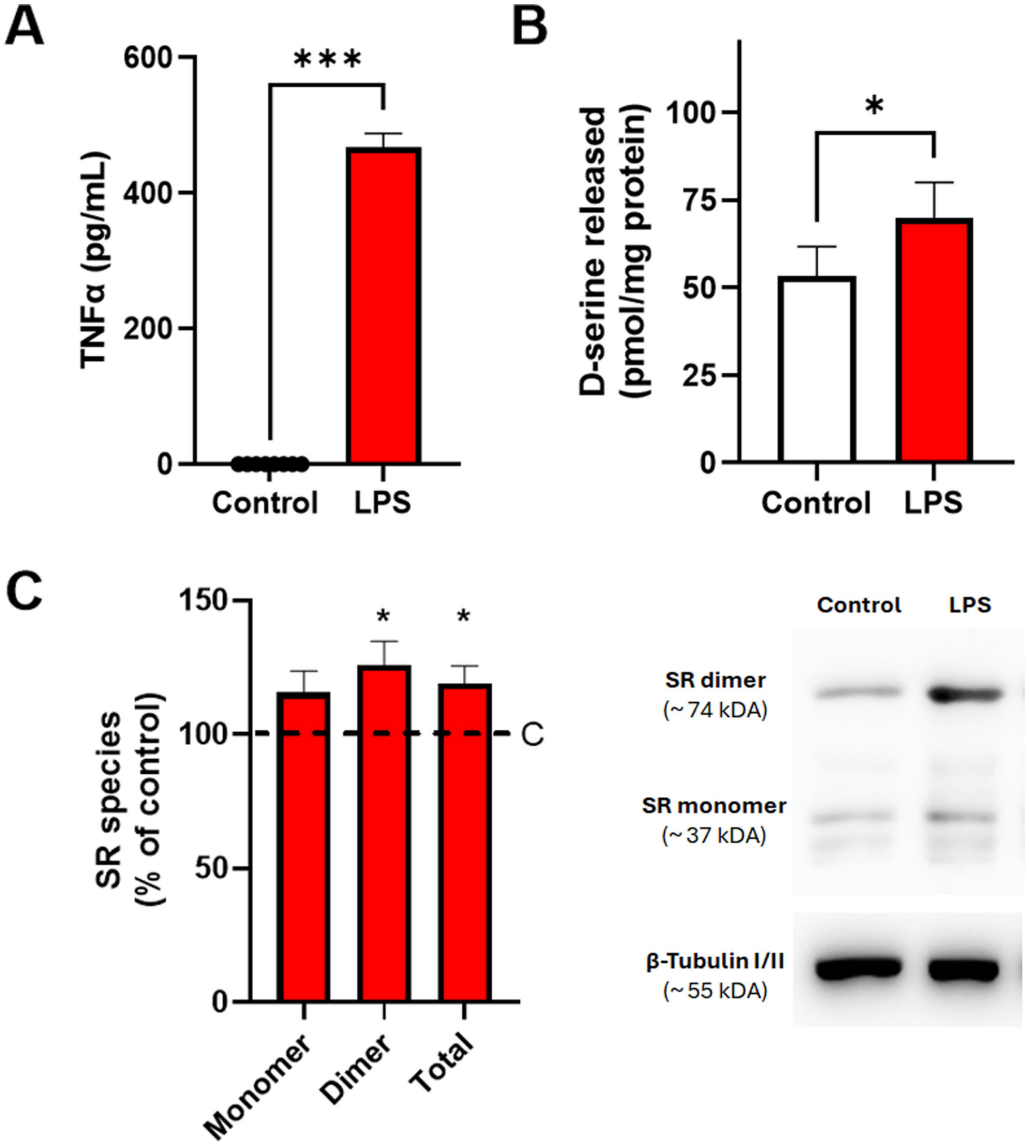
LPS stimulation increases TNFα release and modulates D-serine metabolism in mixed glial cultures. **(A)** TNFα levels (pg/mL) in the culture medium, measured by ELISA 18 h after a 1 h stimulation with LPS. **(B)** D-serine concentration in the extracellular medium, assessed by HPLC and normalized to total protein content (pmol/mg protein). **(C)** Abundance of serine racemase (SR) monomer (~37 kDa), dimer (~74 kDa), and total SR protein levels, quantified by western blot and expressed as percentage of control (**C**, dashed line, 100%). A representative blot is shown alongside the graph. Data represent mean ± SEM from seven independent cultures derived from pooled neonatal cortices. Statistical comparisons were performed using the Wilcoxon matched-pairs signed-rank test. *p < 0.05; ***p < 0.001.

To confirm that mixed glial cultures mounted an inflammatory response to LPS, TNFα levels were measured in the culture medium following stimulation. While TNFα was undetectable under control conditions, LPS-treated cultures showed a marked increase, reaching 467.1 ± 59.5 pg/mL (Figure 4A). In parallel, LPS exposure increased the abundance of serine racemase (SR), as reflected by elevated levels of both monomeric and dimeric SR species (Figure 4C). SR is known to form homodimers, which represent its catalytically active configuration, whereas stable SDS-resistant dimers detected under denaturing conditions have been associated with reduced enzymatic activity (Wang and Barger, 2011; Beltrán-Castillo et al., 2020). Importantly, extracellular D-serine levels in the culture medium were significantly increased following LPS treatment, reaching 135.8% ± 12.8% relative to control values (Figure 4B). Under our experimental conditions (6-well plate format, 900 μL conditioned media per well, and approximately 1 mg total protein per well), these values correspond to estimated bulk extracellular concentrations in the conditioned medium of approximately 0.059 ± 0.009 μM under basal conditions and 0.078 ± 0.011 μM following LPS stimulation. These results indicate that inflammatory stimulation with LPS is accompanied by increased SR abundance and enhanced D-serine release in mixed glial cultures.

### 3.5 Conditioned media from LPS-stimulated glial cultures mimics the effects of D-serine on OPC maturation

To determine whether factors released by glial cells upon inflammatory stimulation—specifically D-serine—modulate OPC maturation, primary OPC cultures were treated with conditioned media (CM) obtained from mixed glial cultures exposed to LPS. CM was collected 18 h after a 1 h LPS stimulation. In selected conditions, D-serine was enzymatically degraded during CM collection by addition of D-amino acid oxidase (DAAO). In parallel, OPC cultures were treated with control media in the presence of the NMDAR antagonist MK-801.

**Figure 5:**
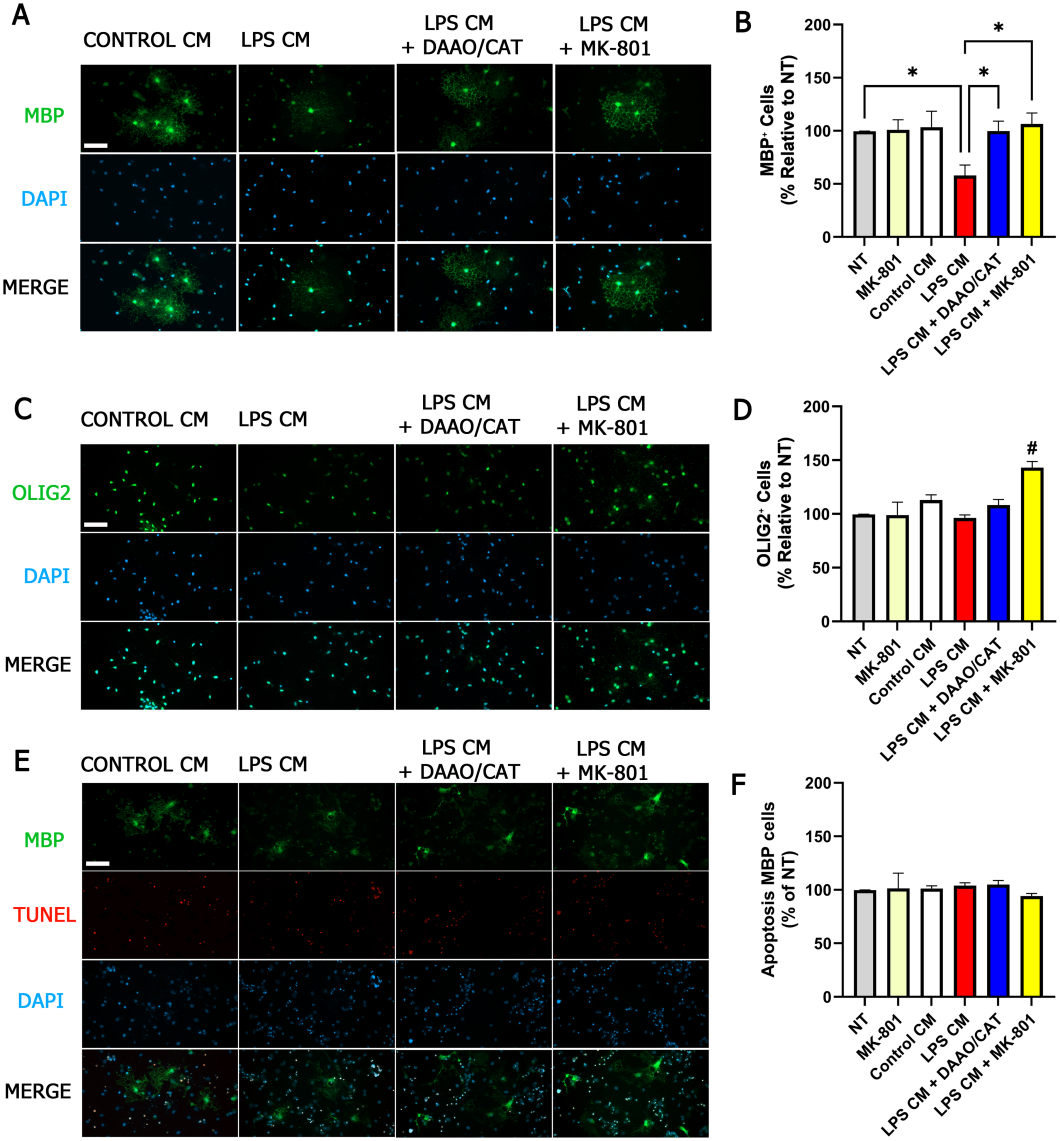
Conditioned media from LPS-stimulated glial cultures modulate OPC differentiation in a D-serine- and NMDAR-dependent manner. Representative immunofluorescence images illustrate MBP **(A)**, OLIG2 **(C)**, and combined MBP/TUNEL staining **(E)** in OPC cultures treated with conditioned media (CM) derived from mixed glial cultures exposed to LPS for 1 h, with or without enzymatic degradation of D-serine using DAAO/catalase (DAAO/CAT) or NMDAR antagonism with MK-801. DAPI was used to label nuclei. All images were acquired using identical imaging settings across conditions. Scale bar: 50 μm (applies to panels **A**,**C**, and **E**). **(B)** Percentage of MBP^+^ cells in OPC cultures. **(D)** Percentage of OLIG2^+^ cells. **(F)** Apoptotic MBP^+^ cells quantified by TUNEL staining. For **(F)**, data are normalized to the mean value observed in the NT group (non-treated OPC cultures maintained without conditioned media; 14.9% ± 1.6% of total cells), and no significant differences were detected across treatments. Data are presented as mean ± SEM from 6 to 9 independent cultures. Statistical comparisons were performed using the Kruskal–Wallis test followed by Dunn’s *post hoc* test. *p < 0.05; #*p* < 0.05 compared to NT.

Exposure to CM derived from LPS-stimulated glial cultures resulted in a significant reduction in the proportion of MBP^+^ cells (Figures 5A,B), qualitatively resembling the effects observed following direct D-serine treatment. Notably, this effect was no longer observed when D-serine was enzymatically removed from the CM or when OPCs were co-treated with the NMDAR antagonist MK-801. In addition, co-treatment with MK-801 prevented the reduction in MBP^+^ cells induced by LPS-conditioned media and was associated with an increased proportion of OLIG2^+^ cells (Figures 5C,D). Importantly, treatment with MK-801 alone did not significantly alter total cell number, apoptosis rates, or the proportion of OLIG2^+^ or MBP^+^ cells compared to control conditions (Figures 5B,D,F). Apoptosis levels within the MBP^+^ population remained unchanged across all conditions (Figures 5E,F), suggesting that the observed effects primarily reflect altered oligodendroglial maturation rather than changes in cell survival.

## 4 Discussion

In this study, we show that D-serine functions as a glia-derived signal capable of modulating oligodendrocyte lineage progression under inflammatory conditions. Exposure of OPC cultures to D-serine during a late differentiation window (DIV6–DIV7), corresponding to a transitional stage preceding the marked expansion of MBP^+^ cells observed at DIV8, reduced the proportion of OLIG2^+^ and MBP^+^ cells without affecting net cell number, while selectively decreasing apoptosis within the mature MBP^+^ population. Consistent with these observations, conditioned media derived from LPS-stimulated mixed glial cultures reproduced the effects of D-serine exposure on OPC maturation, leading to a reduction in MBP^+^ cells. Importantly, this effect was no longer observed following enzymatic degradation of D-serine or by pharmacological blockade of NMDARs with MK-801. Across these experimental paradigms, D-serine exposure was associated with a redistribution of lineage-marker expression, characterized by reduced OLIG2^+^ and MBP^+^ fractions and a corresponding increase in the OLIG2^−^/MBP^−^ category. Although the reduction in MBP^+^ cells under LPS-conditioned media was of comparable order to that induced by direct D-serine exposure, the absence of a replicated anti-apoptotic effect suggests that additional soluble mediators released during glial activation contribute to the overall phenotype. LPS stimulation generates a complex inflammatory milieu that includes pro- and anti-inflammatory cytokines and reactive oxygen species (McTigue and Tripathi, 2008; French et al., 2009; Miron et al., 2013; Miron, 2017). In this context, D-serine likely represents one component of a broader inflammatory signaling network influencing oligodendroglial lineage progression. Accordingly, while our data support a significant contribution of D-serine–dependent NMDAR signaling, additional inflammatory mediators present in conditioned media may engage parallel or interacting pathways that influence OPC maturation. In addition, D-serine has been reported to influence serine palmitoyltransferase (SPT) activity and sphingolipid metabolism in certain contexts (Hanada et al., 2000), indicating that receptor-independent effects cannot be entirely excluded. Nevertheless, within the CNS its most extensively characterized function remains NMDAR co-agonism, and the reversal of the observed effects by both NMDAR blockade and enzymatic degradation of D-serine in our system is consistent with a receptor-mediated mechanism. Taken together, these findings are consistent with a significant contribution of D-serine–dependent NMDAR signaling in linking inflammatory glial activation to altered oligodendrocyte lineage progression under our experimental conditions.

Inflammatory activation of glial cells provides a context in which D-serine may influence oligodendrocyte lineage dynamics. We show that LPS exposure induced a reactive glial state characterized by robust TNFα release, increased serine racemase expression, and elevated extracellular D-serine levels. These findings are consistent with previous reports demonstrating inflammation-induced D-serine production in glial cells (Wu et al., 2004, 2007; Beltrán-Castillo et al., 2020) and support the concept of D-serine as a non-classical gliotransmitter operating within inflammatory microenvironments. Within this inflammatory setting, glia-derived D-serine emerges as a candidate signal capable of modulating oligodendroglial lineage progression. Bulk conditioned-media measurements in our system fall within the tens-of-nanomolar range. However, these values were normalized to total protein content and therefore reflect relative D-serine production rather than the free extracellular concentration at the cell surface. Accordingly, bulk measurements may not capture transient or spatially restricted extracellular gradients. *In situ* biosensor studies have reported low-micromolar increases in extracellular D-serine under activity-dependent conditions (Van Horn et al., 2017), supporting the rationale for probing a low-micromolar functional range *in vitro*.

In the present study, inflammatory signaling was examined using mixed glial cultures containing both astrocytes and microglia, which more closely recapitulate cellular interactions present in inflammatory CNS environments. Under these conditions, LPS exposure increased serine racemase immunoreactivity across both monomeric (~37 kDa) and SDS-resistant dimeric (~74 kDa) species detected under denaturing conditions (Wu and Barger, 2004; Wu et al., 2004, 2007; Wang and Barger, 2011), consistent with an overall increase in SR abundance rather than a selective shift between structural species. This increase paralleled elevated extracellular D-serine levels in conditioned media, consistent with increased D-serine availability within the glial microenvironment. While this experimental design does not allow direct attribution of SR regulation to a single glial population, it reflects the integrated response of glial networks under inflammatory conditions, differing from reports in pure microglial cultures showing context-dependent regulation of SR species (Beltrán-Castillo et al., 2020).

Within this inflammatory context, the selective reduction of MBP^+^ oligodendrocytes in the absence of increased apoptosis within the oligodendrocyte lineage suggests that the effects of D-serine on oligodendrocyte maturation are unlikely to reflect generalized cytotoxicity and instead may reflect stage-dependent differences in NMDAR-related outcomes across the oligodendrocyte lineage. The coexistence of reduced apoptosis within the MBP^+^ population alongside diminished progression of immature cells toward OLIG2^+^ or MBP^+^ states support the possibility that inflammatory cues, such as glia-derived D-serine, differentially regulate survival and maturation within the oligodendrocyte lineage. NMDAR activation is known to produce context- and intensity-dependent outcomes, ranging from excitotoxicity to pro-survival signaling depending on receptor composition, calcium dynamics, and intracellular coupling mechanisms (Káradóttir et al., 2005; Micu et al., 2006; Li et al., 2013). Within late-stage oligodendrocytes, moderate D-serine–dependent NMDAR activation may engage survival-associated pathways without promoting terminal differentiation, potentially decoupling maturation from viability under inflammatory conditions.

In this context, our observation that oligodendrocyte maturation is reduced in the absence of increased cell death is consistent with the possibility that D-serine–dependent NMDAR signaling operates outside the pro-differentiation range previously described under physiological, non-inflammatory conditions (Li et al., 2013). In rat OPC cultures, NMDA stimulation enhances myelin protein expression and process branching during late differentiation, increasing the number of MBP^+^ oligodendrocytes through mechanisms linked to mTOR signaling (Guardiola-Diaz et al., 2012; Li et al., 2013). These findings indicate that NMDAR signaling per se plays an intrinsic role in regulating oligodendroglial differentiation, independent of inflammatory D-serine signaling. Importantly, our findings do not contradict these observations but instead highlight the strong context dependence of NMDAR function across the oligodendroglial lineage. Consistent with this view, Li et al. (2013) described a biphasic response to NMDA, whereby moderate concentrations (100 μM) promoted OPC differentiation, whereas higher concentrations (1,000 μM) reduced MBP^+^ cell numbers without affecting survival. In addition, the functional diversity of NMDARs is underscored by the existence of receptor subtypes, such as GluN1/GluN3 assemblies, which can be activated by D-serine in the absence of glutamate, indicating that receptor composition may confer distinct functional properties to D-serine–dependent responses (Piña-Crespo et al., 2010). In this interpretative framework, differences in NMDAR subunit composition across oligodendroglial lineage stages may contribute to, rather than directly determine, whether D-serine signaling promotes differentiation, limits progression toward terminal MBP^+^ maturation, or supports survival without maturation.

Consistent with this interpretation centered on late-stage differentiation, electrophysiological studies have demonstrated that NMDAR-mediated responses differ markedly across oligodendroglial lineage stages. NMDA-evoked currents are present in oligodendrocyte lineage cells but exhibit distinct amplitudes and biophysical properties as cells progress toward more mature phenotypes, suggesting that NMDAR signaling can engage different functional programs depending on the differentiation context (Káradóttir et al., 2005). Within the late differentiation window examined in the present study, this functional diversity provides a physiological framework in which D-serine–dependent NMDAR signaling under inflammatory conditions may bias lineage-marker expression, potentially limiting progression toward terminal MBP^+^ maturation while supporting survival of the MBP^+^ population.

Given the stage-dependent NMDAR outcomes described above, inflammatory D-serine signaling may contribute to altered oligodendrocyte lineage progression in inflammatory environments, limiting terminal differentiation without necessarily increasing oligodendroglial cell death. This interpretation aligns conceptually with observations in chronic multiple sclerosis lesions, where oligodendrocyte lineage cells extend processes toward demyelinated axons but fail to form compact myelin sheaths (Chang et al., 2002). In this setting, glia-derived D-serine may contribute to persistence of a non-myelinating oligodendrocyte state, without necessarily implying a fully adaptive or maladaptive outcome. Rather than acting as an irreversible inhibitor of oligodendrocyte maturation, D-serine signaling may function as a temporal checkpoint-like mechanism that delays terminal differentiation under inflammatory conditions. Although the present study does not directly address therapeutic modulation, these findings raise the possibility that modulation of D-serine metabolism could represent a future avenue to rebalance oligodendrocyte survival and differentiation under inflammatory conditions. Thus, D-serine signaling emerges as a modulatory factor linking inflammatory status to the temporal regulation of oligodendrocyte differentiation, rather than as a purely detrimental or protective signal.

Several limitations of this study should be acknowledged. First, although our OPC cultures exhibited a high degree of purity (>99.0%), indirect contributions from the small fraction of contaminating glial cells cannot be completely excluded. D-serine or other bioactive components present in conditioned media could theoretically act on residual astrocytes or microglia, which might in turn influence OPC maturation through secondary signaling mechanisms. We did not perform a dedicated post-treatment quantification of GFAP^+^ or IBA1^+^ cells following D-serine exposure; however, no obvious qualitative increase in non-oligodendroglial cells was observed under these conditions. Nevertheless, the minimal presence of astrocytes and microglia, together with the reproducibility of the effects observed following both direct D-serine application and conditioned media treatments, is consistent with a predominantly oligodendroglial effect of D-serine under our experimental conditions. Second, our inference of NMDAR involvement is based on pharmacological blockade using the non-competitive antagonist MK-801, which does not provide lineage-specific resolution. Although the high purity of our cultures and the consistency of the effects following direct D-serine exposure reduce the likelihood of indirect mechanisms, definitive attribution to oligodendrocyte-lineage NMDARs will require future studies employing genetic or cell-type–restricted approaches. In addition, while OLIG2 and MBP allowed us to assess lineage progression, precise resolution of the OLIG2^−^/MBP^−^ fraction into specific oligodendroglial sub-states would require additional stage-specific markers and was beyond the scope of the present study. Third, the experimental design does not allow discrimination of the relative contributions of astrocytic versus microglial serine racemase (SR) activity to the observed increase in D-serine under inflammatory conditions. Although both cell types are known to express SR and upregulate D-serine production during inflammation, they may exert distinct influences on OPC biology that cannot be resolved with the approaches used here (Miron et al., 2013; Miron, 2017). Astrocytes represent a major source of D-serine under physiological conditions (Wolosker et al., 1999a, 1999b; Beltrán-Castillo et al., 2017), whereas activated microglia release D-serine alongside proinflammatory cytokines (Wu et al., 2007; Tapanes et al., 2022), potentially amplifying its impact on oligodendrocyte lineage progression. Dissecting these cell-type–specific contributions will be an important goal of future studies. Finally, our findings are derived from *in vitro* culture systems and should be interpreted within this context. Although LPS provides a robust and well-characterized stimulus to induce glial activation via TLR4 signaling, it represents a simplified inflammatory paradigm. The inflammatory environment in demyelinating diseases such as multiple sclerosis involves complex cytokine networks, adaptive immune components, and chronic lesion evolution that are not fully recapitulated by LPS exposure. Nevertheless, the robust induction of TNFα and D-serine observed in our model supports its relevance for dissecting inflammation-associated glial signaling mechanisms under controlled conditions. Thus, while extrapolation to complex disease settings should be approached cautiously, our findings provide mechanistic insight into how inflammation-associated D-serine signaling may influence oligodendrocyte lineage dynamics.

## 5 Conclusion

This study provides evidence that inflammation-associated release of D-serine from glial cells modulates oligodendrocyte lineage progression in a manner sensitive to NMDAR blockade. Elevated D-serine levels selectively restrain oligodendrocyte differentiation while preserving the survival of more mature cells, supporting a dissociation between lineage progression and cell viability under inflammatory environments. Together, our findings support a role for D-serine as a molecular link between glial inflammatory activation and altered oligodendrocyte maturation *in vitro*, providing mechanistic insight into how inflammation-associated signaling may influence remyelination dynamics.

## Data Availability

The raw data supporting the conclusions of this article will be made available by the authors, without undue reservation.
